# The Univariate Flagging Algorithm (UFA): An interpretable approach for predictive modeling

**DOI:** 10.1371/journal.pone.0223161

**Published:** 2019-10-11

**Authors:** Mallory Sheth, Albert Gerovitch, Roy Welsch, Natasha Markuzon

**Affiliations:** 1 Massachusetts Institute of Technology, Cambridge, Massachusetts, United States of America; 2 The Charles Stark Draper Laboratory, Cambridge, Massachusetts, United States of America; Ben-Gurion University of the Negev, ISRAEL

## Abstract

In many data classification problems, a number of methods will give similar accuracy. However, when working with people who are not experts in data science such as doctors, lawyers, and judges among others, finding interpretable algorithms can be a critical success factor. Practitioners have a deep understanding of the individual input variables but far less insight into how they interact with each other. For example, there may be ranges of an input variable for which the observed outcome is significantly more or less likely. This paper describes an algorithm for automatic detection of such thresholds, called the Univariate Flagging Algorithm (UFA). The algorithm searches for a separation that optimizes the difference between separated areas while obtaining a high level of support. We evaluate its performance using six sample datasets and demonstrate that thresholds identified by the algorithm align well with published results and known physiological boundaries. We also introduce two classification approaches that use UFA and show that the performance attained on unseen test data is comparable to or better than traditional classifiers when confidence intervals are considered. We identify conditions under which UFA performs well, including applications with large amounts of missing or noisy data, applications with a large number of inputs relative to observations, and applications where incidence of the target is low. We argue that ease of explanation of the results, robustness to missing data and noise, and detection of low incidence adverse outcomes are desirable features for clinical applications that can be achieved with relatively simple classifier, like UFA.

## Introduction

Classifiers can be evaluated by multiple parameters, including accuracy, robustness, sensitivity to missing data, or ease of interpretability. Good predictive accuracy is often by far the most important evaluation metric. However, many practitioners are hesitant to use machine learning classifiers as they struggle to explain the results and understand a classifiers ‘reasoning’ behind a solution. In the analysis of clinical data, this hesitation is particularly pronounced–doctors need to be able to interpret the prognosis and analyze which of the variables and their combination led to a prediction.

Donoho and Jin [1] have demonstrated that the use of very simple univariate discriminant analysis, making no use of covariance matrices, led to a similar performance on a standard series of datasets [2] compared to much more sophisticated popular machine learning methods (including Boosted decision trees, Random Forests, SVM, KNN, PAM and DLDA). The authors argue that whenever useful, such methods provide better explanatory power and visibility of contributing factors than more complex methods. Similarly, authors of the Mas-o-Menos algorithm [3] compared their simplified approach to more sophisticated algorithms for treatment predictions of bladder, breast, and ovarian cancers, and came to the conclusion that model interpretation and validation were more important than complexity.

A number of recent studies focused on building predictive models that are highly accurate, and yet also highly Interpretable. One of the proposed solutions includes decision sets of independent if-then rules, which can be applied independently [4]. Such a univariate approach allows easy interpretation of the results, and has been demonstrated to achieve high accuracy.

Many nonlinear classifiers, such as Decision Trees [5] or Support Vector Machines (SVM) [6], are designed to find “optimal” cutpoints, typically defined as cutpoints that minimize some measure of node impurity. Such measures include misclassification rate, Gini index, or entropy/information gain [5, 6]. Supervised clustering works similarly, minimizing impurity while adding a penalty for the total number of clusters [7]. Alternatively, Williams, et al (2006) put forth a minimum p-value approach for finding optimal cutpoints for binary classification [8]. Their algorithm uses a chi-squared test to find the cutpoint that maximizes the difference in outcomes between both sides.

These approaches are similar in that they consider the entire input space, both false positives and false negatives, to select the optimal cutpoint. In certain applications, however, one may care more about a subspace of increased incidence of a target. Under certain conditions, it might be important to identify separation thresholds that are associated with a high prevalence of the target, while the overall solution is not optimized. Examples include medical conditions where values outside clinically defined thresholds are associated with high mortality, while more normal values may not provide much information. In clinical decision making, doctors identify ranges of laboratory tests values that may identify patients’ higher risk of developing or having a disease [8, 9]. In earth science, amount of rainfall thresholds can be used to develop early warning systems for landslides or flooding [10, 11].

**Fig 1:**
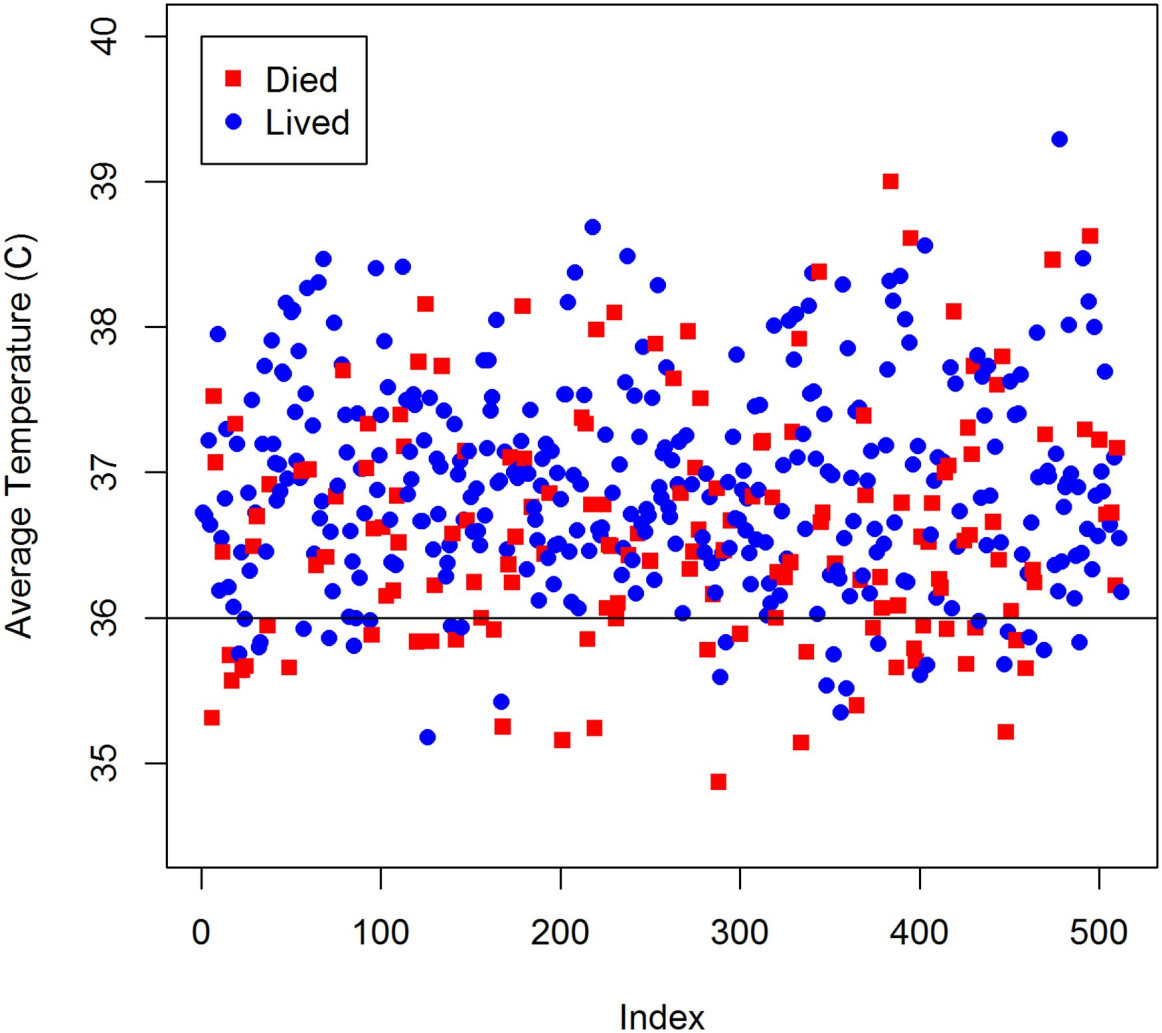
Body temperature for adult sepsis patients.

For example, in individuals with sepsis, low body temperature is associated with illness severity and death [12]. Fig 1 displays average body temperature for 512 septic patients, with an overall death rate of 30.9%. Patients who died are denoted in red, while patients who survived are denoted in blue. International guidelines for sepsis management define low body temperature as 36° C [13]. For patients below this threshold the death rate stands at 57.1%, nearly twice the overall death rate, while little can be said about patients above the threshold (Fig 1).

We propose an algorithm for identifying such thresholds in an automated fashion. In the decision tree or SVM framework, cost functions penalizing for false positives or negatives will shift the “optimal” cutoff to optimize the cost or other criterion. [5, 6]. In practice, however, it is often difficult to quantify the costs associated with different types of errors, in particular in the medical domain.

Friedman & Fisher’s (1999) Patient Rule Induction Method (PRIM) procedure finds rectangular subregions of the feature space that are associated with a high (or low) likelihood of the outcome. The subregions are then slowly made smaller, each time increasing (or decreasing) the rate of the outcome [14]. With this method and others like it, there is an inherent tradeoff between the number of data points within the subregion (the support) and the proportion of the data points that are associated with the outcome (purity), where smaller supports generally have higher purity. With PRIM, the user is responsible for defining the “optimal” subregion, by specifying the preferred trade off for the application. While this may work well in some situations, identifying the appropriate tradeoff is challenging, suggesting the need for an algorithm that requires less user input.

We decided to consider each explanatory variable separately as in Donoho and Jin [1]. In line with expectations of interpretability for methods to be adopted and used by emergency room medical professionals, we aimed to avoid using correlation between variables and to try to find methods more related to how doctors evaluate individual explanatory variables, and only then treat them as a group. Since doctors tended to notice the very high and/or very low values of each explanatory variable, we developed methods to relate very high and/or low values to the chances of surviving or dying. The result is the flagging algorithm, called the Univariate Flagging Algorithm (UFA), which directly relates the higher or lower values of an explanatory variable to chances of survival or death and is easily understood.

UFA optimizes over subregions of the input space but performs the tradeoff between support and purity in a well-defined way. We show that UFA can identify the existence of thresholds for individual variables. We demonstrate that these thresholds can be used to classify previously unseen test cases with performance equal to or better than many commonly used classification techniques when confidence intervals are considered. The surprising accuracy in predictive power of such an approach is also boosted by a good tolerance to noise and missing data. With each variable treated independently, there is no need to compute complex interaction parameters that are sensitive to missing data. In clinical data analyses, data imputations are not desirable and this feature of the proposed algorithm becomes attractive.

## Methods

UFA is designed to identify an optimal cutpoint for a single explanatory variable that is associated with a significantly higher or lower likelihood of the target. UFA identifies up to two such thresholds, one below the median and one above the median. The algorithm is intended for a binary or continuous target *y* (e.g. [0, 1]) and a continuous explanatory variable. At its most basic level, UFA finds the value *x* = *x*^*opt*^ that maximizes the difference in the outcome rate for observations that fall outside *x*^*opt*^ and a baseline rate, while maintaining a good level of support.

### Formal specification

The following variables are necessary for the formal specification of the UFA algorithm (Table 1). For the purpose of formulation, we consider candidate thresholds below the median value of *x*.

**Table 1:** List of variables for specification of UFA algorithm. For the purpose of formulation, we consider candidate thresholds below the median value of *x*.

Variable	Definition
*y* ∈ {0, 1}	Binary target
*x* ∈ [*x*_*min*_, *x*_*max*_]	Continuous explanatory variable
*x*_*iqr*_ ∈ [*x*_*P*25_, *x*_*P*75_]	Values of *x* in the interquartile range, defined as 25^th^ to 75^th^ percentile
*n*_*iqr*_	Number of observations in *x*_*iqr*_
*p*_*iqr*_	Percent of *n*_*iqr*_ with *y* = 1
${\hat{x}}_{i}$	Candidate threshold below median of *x*
*n*_*i*−_	Number of observations below candidate threshold
*p*_*i*−_	Percent of *n*_*i*−_ with *y* = 1

For each ${\hat{x}}_{i}$, we conduct the following hypothesis test to check for a significant difference in the outcome rate below the threshold and the outcome rate in the interquartile range:

$$\begin{array}{l} H_{0}:p_{i-}-p_{iqr}=0\\ H_{a}:p_{i-}-p_{iqr}\neq 0 \end{array}$$ (1)

We are using a binomial proportion test [15] with test statistic *Z*_*i*_:

$$Z_{i}=\frac{p_{i-}-p_{iqr}}{\sqrt{p_{i-}^{wa}*\left(1-p_{i-}^{wa}\right)*\left(\frac{1}{n_{iqr}}+\frac{1}{n_{i-}}\right)}}$$ (2)

where $p_{i-}^{wa}$ is the weighted average of the outcome rates, calculated:

$$p_{i-}^{wa}=\frac{\left(p_{iqr}*n_{iqr}+p_{i-}*n_{i-}\right)}{n_{iqr}+n_{i-}}$$ (3)

We define ${\hat{x}}^{opt}$ as the candidate threshold ${\hat{x}}_{i}$ with the maximum *Z*_*i*_ in absolute value:

$${\hat{x}}^{opt}=\underset{{\hat{x}}_{i}}{\text{max}}\left[abs\left(Z_{i}\right)\right]$$ (4)

*Z*_*i*_ provides an inherent trade-off between providing a chosen support level and maximizing (or equivalently, minimizing) the outcome rate. The proposed measure does not minimize the overall misclassification rate; instead it is designed to identify areas enriched with the target outcome cases. The same applies to finding areas with a specifically low rate of the target.

#### Procedure to find optimal threshold for variable *x*

1. Generate a list of potential thresholds ${\hat{x}}_{i-}$ between the median value of *x* and the minimum value of *x*, excluding those with low support.
  - For the purpose of this paper, we excluded the five lowest values of *x*, assuming that thresholds with a support of fewer than five are of no interest. In future versions we will reconsider this assumption in order to include possibly outlying observations.
  - Currently, we identify potential thresholds ${\hat{x}}_{i-}$ by dividing the range of values between the median and minimum value of *x* into 50 segments of equal length. Future research should evaluate whether a more sophisticated method for selecting possible thresholds can further improve the performance of UFA.
2. Calculate *Z*_*i*_ as specified in Eq (2). Define ${\hat{x}}^{opt}$ according to Eq (4).
3. Check ${\hat{x}}^{opt}$ for statistical significance by comparing its Z-value to a chosen critical value. Keep the threshold if it is significant and discard it otherwise.
  - For the purpose of this paper, we used a critical value of 2.576 to establish significance, which is associated with a p-value of 0.01. We address issues related to multiple testing by validating the thresholds on previously unseen data.

Through this procedure, UFA finds the near-optimal threshold below the median for each variable *x*. The procedure can then be repeated for area above the median.

UFA is a univariate algorithm that does not take into account interactions between variables. However, known interactions can be incorporated into the UFA framework by introducing new variables that are combinations of existing features. Some other single variable approaches are contained in Donoho and Jin [1]. We found our approach to be especially useful and transparent in medical settings.

### Classification using UFA

There are many approaches to incorporate UFA-designed thresholds into a multi-dimensional classifier, and we present two possibilities in this paper. Both create an indicator variable or “flag” for each significant threshold, which takes the value of one if the value of the variable under consideration exceeds the threshold and zero otherwise.

Given the flags on each of hundreds of explanatory variables, it is necessary to develop a scoring mechanism that is as transparent as possible. Since we had two dimensions (survival and mortality), it is natural to make a scatterplot of the number of mortality flags against the number of survival flags for the training data and then denote which patients survived to day four and which had died by day four. When data from the second day is available for a new patient, the doctor can see where the patient is located on the scatterplot. There are many possible ways to draw some form of boundary for a decision to be made about the chances of the new patient surviving to day four. Again, thinking about visual transparency, we decided on a linear boundary based on linear discriminant function analysis. Other methods are clearly possible (support vector machines, near-neighbors, etc.) but are harder to explain or lead to complex boundaries or both.

#### Number of Flags algorithm (N-UFA)

**Fig 2:**
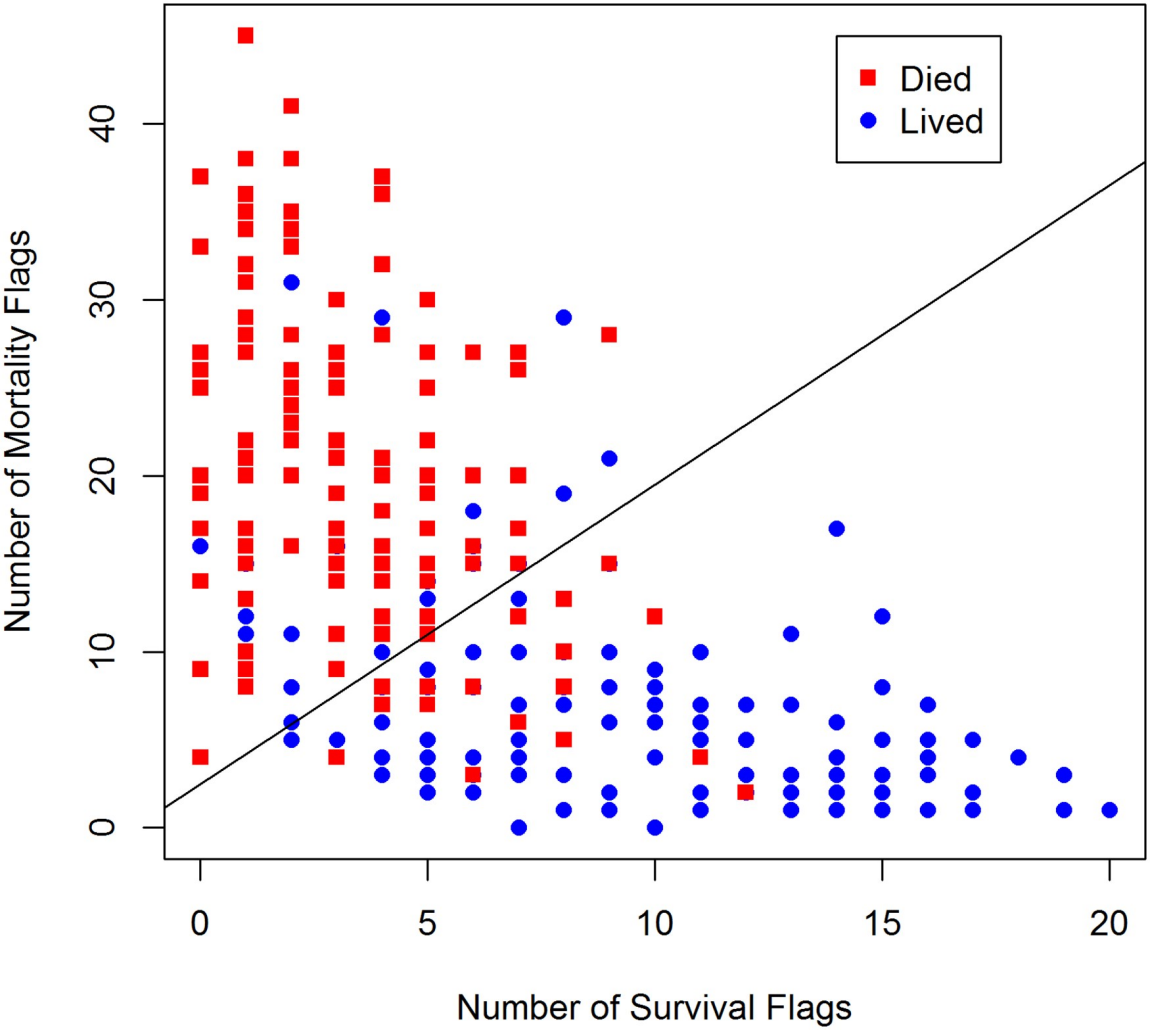
Number of high mortality and low mortality flags for adult sepsis patients. Patients who died are indicated by red squares while patients who lived are indicated by blue triangles. For each patient, we counted the number of flags that are associated with a high likelihood of mortality and the number of flags that are associated with a low likelihood of mortality; the solid line represents a prototype of a linear decision boundary that would be designed to minimize the misclassification rate along these two dimensions.

The first classifier aggregates the number of “high risk” and “low risk” flags for each observation, creating a two-dimensional vector for each observation. Then, a linear decision boundary is drawn to separate one class from the other along these two dimensions. Throughout the paper, this approach will be denoted as the Number of Flags algorithm (N-UFA). Fig 2 shows an example of the N-UFA classifier’s performance in predicting adult sepsis patients’ mortality. For each patient, we count the number of flags that are associated with a high likelihood of mortality and the number of flags that are associated with a low likelihood of mortality; the solid line represents the linear decision boundary that minimizes the misclassification rate along these two-dimensions. Throughout this paper, each flag receives an equal weight of one, though future research could investigate the impact of assigning flags different weights.

#### UFA-created thresholds as features in Random Forest (RF-UFA)

The second classifier uses UFA-identified flags as independent dummy features in a Random Forest model [5].

## Results

In this section, we apply the UFA system to a variety of different datasets and compare its performance to other commonly used classification techniques. We present results for datasets that vary greatly in terms of complexity and target/non-target ratio, allowing us to identify conditions for which UFA is particularly well suited.

### Benchmark datasets (Pima Diabetes, Wisconsin Breast Cancer, ALL/AML Cancer)

Table 2 summarizes the performance of UFA for three well-known classification datasets. First two datasets have relatively few attributes, little to no missing data, and a target/non-target ratio of approximately one-third [16, 17]. For these datasets, the performance of UFA evaluated using the out-of-sample error is in line with published results for other standard classifiers [18, 19] and shows a similar or better performance in our experiments. The third dataset was selected to evaluate UFA performance without any customization when the number of independent variables is large. UFA has demonstrated excellent recognition accuracy on the ALL/AML cancer dataset [20,21] that contains 7,167 independent variables (Table 2). The reported results are the average of 1000 independent experiments performed for each setting.

**Table 2:** Comparison of error rates on previously unseen data. Results show out of sample error rate averaged over 1000 runs.

Algorithm	Pima Indian Diabetes	Wisconsin Breast Cancer	ALL/AML Cancer
N-UFA	18.2 ± 2.2	6.8 ± 1.6	0.9 ± 1.9
R-UFA	19.3 ± 2.3	7.2 ± 1.6	0.6 ± 1.6
Logistic Regression	23.0 ± 2.3	5.8 ± 1.9	44.9 ± 12.9
Random Forest	24.1 ± 2.5	4.1 ± 1.4	5.9 ± 5.8
Conditional Inference Tree	26.1 ± 3.0	6.7 ±1.9	9.9 ± 7.0
SVM	24.1 ± 2.4	2.7 ± 1.2	20.6 ± 11.4
k-NN	30.3 ± 2.5	7.5 ± 1.7	8.8 ± 5.5

Evaluated on robustness to missing data, UFA has shown a similar or better performance as compared to a set of standard classifiers (Table 3).

**Table 3:** Robustness to missing data. Results show out of sample error rate averaged over 1000 runs.

	Full Set	10% Missing	50% Missing
**Pima Indian Diabetes**			
N-UFA	18.2 ± 2.2	24.6 ± 2.4	28.0 ± 2.6
R-UFA	19.3 ± 2.3	26.7 ± 2.7	28.2 ± 2.7
Logistic Regression	23.0 ± 2.3	27.3 ± 2.7	33.7 ± 2.6
Random Forest	24.1 ± 2.5	25.1 ± 2.5	31.5 ± 2.6
Conditional Inference Tree	26.1 ± 3.0	27.1 ± 2.9	33.6 ± 3.0
SVM	24.1 ± 2.4	24.7 ± 2.6	32.8 ± 2.6
k-NN	30.3 ± 2.5	31.0 ± 2.6	33.9 ± 2.6
**Wisconsin Breast Cancer**			
N-UFA	6.8 ± 1.6	7.0 ± 1.7	6.8 ± 1.8
R-UFA	7.2 ± 1.6	6.4 ± 1.6	6.2 ± 16
Logistic Regression	5.8 ± 1.9	8.1 ± 2.1	16.3 ± 2.4
Random Forest	4.1 ± 1.4	4.8 ± 1.6	7.4 ± 1.9
Conditional Inference Tree	6.7 ±1.9	8.8 ± 2.4	13.0 ± 2.5
SVM	2.7 ± 1.2	6.3 ± 1.8	12.4 ± 2.2
k-NN	7.5 ± 1.7	9.2 ± 1.8	13.3 ± 2.4
**ALL/AML Cancer**			
N-UFA	0.9 ± 1.9	0.8 ± 1.8	0.0 ± 0.1
R-UFA	0.6 ± 1.6	0.8 ± 1.7	0.0 ± 0.0
Random Forest	5.9 ± 5.8	9.8 ± 8.7	27.2 ± 9.8
Conditional Inference Tree	9.9 ± 7.0	11.7 ± 7.3	34.3 ± 8.8
SVM	20.6 ± 11.4	27.6 ± 13.8	35.0 ± 8.3
k-NN	8.8 ± 5.5	18.5 ± 9.8	31.7 ± 8.8

### Missing and noisy data (MIMIC II)

The publicly available MIMIC II database, version 2.6, contains de-identified clinical data for over 30,000 adult intensive-care unit (ICU) stays [22]. Focusing on patients admitted with a primary diagnosis of sepsis, we processed over 200 variables covering the first four days of the patient’s stay. These variables included both static features like demographics, as well as dynamic features such as trends in laboratory values or vital signs. As outlined in the introduction, our final dataset contained 512 patients with a mortality rate of 30.9%. In addition to having a much larger number of attributes than the datasets presented in the last section, the MIMIC II data comes directly from patients’ medical records and is subject to missing and noisy values. More than 65% of the patients in the analysis have incomplete data.

We used the MIMIC II data to evaluate UFA in two different ways. First, we reviewed individual thresholds (${\hat{x}}^{opt}$) identified by the algorithm to determine whether they aligned with known physiological boundaries for those variables for which such boundaries are known (e.g. clinical tests are available). The majority of the created variables did not have such known boundaries, as they were derivatives from a patient’s vital signs or other ICU-recorded measurements. Verifying known values, we extrapolated the assumption that UFA estimates have clinical value to these variables’ boundaries as well.

Our analysis of UFA thresholds (${\hat{x}}^{opt}$) suggests that the algorithm identifies logical break points that align with subject matter expertise. For example, in this paper’s introduction, Fig 1 displays body temperature for patients with sepsis, and includes a cut-point at 36° C aligning with the clinical definition of low body temperature. In sepsis, low body temperature is one of the diagnostic criteria for severe sepsis and septic shock, and is known to be associated with patient severity and death [8]. Applying UFA to the MIMIC II data for body temperature, we identify a high-mortality threshold at 35.97°C which aligns closely with the known physiological limit. Below the threshold of 35.97°C, sepsis patients in the MIMIC II dataset die at a rate of 57.9%, nearly twice the overall death rate.

Table 4 shows three other examples of variables in the MIMIC II database with known clinical thresholds. For each variable, UFA identifies a significant threshold that is well within one standard error of a known bound, as established by the National Institutes of Health [23]. Moreover, though UFA only identified one significant threshold for each variable in Table 4, the directionality is consistent with clinical understanding of sepsis. For example, it is well-known that hypotension, or *lowered* blood pressure, is associated with worsening sepsis [13], which is consistent with our findings.

**Table 4:** Examples of UFA-defined thresholds, MIMIC II data. For each variable in the table, the UFA-identified threshold aligns with the known physiological bound, as established by the National Institutes of Health. The mortality rates for patients who violated these thresholds range from 52.7% to 55.9%, much higher than the 30.9% death rate in the septic population overall.

Variable	Normal Range	Threshold		Support	Mortality
Phosphorus Level	2.4–4.1 mg/dL	More Than	4.5	93	52.7%
Sodium Level	135–145 mEq/L	Less Than	134.9	59	55.9%
Mean Arterial BP	70–110 mmHg	Less Than	67.4	86	55.8%

Next, we used the thresholds identified by UFA to predict mortality in sepsis patients and compared our performance to other commonly used classification techniques. The classifiers contained in Table 4 were implemented in the R computing package, using the built-in tuning parameters [24,25,26,27]. While UFA does not require complete data for each observation to make predictions, many other classifiers do. In these cases, missing values were imputed using the sample average. While other imputation approaches exist, a full survey is outside the scope of this paper.

Using ten-fold cross validation, on average, N-UFA correctly classifies 77.5% of test cases, while RF-UFA achieves 78.1% accuracy. As seen in Table 5, when the confidence intervals are considered, this performance is better than or comparable to classifying patients based on the original, continuous data for a variety of commonly used linear and non-linear methods. Similarly, the AUROC for the two UFA-based classifiers is significantly higher than all of the non-UFA methods with the exception of random forest but overlaps RF when the confidence intervals are taken into account.

**Table 5:** Comparison of different classifiers for in-hospital mortality of adult sepsis patients. The two UFA-based classifiers have predictive performance better than or equal to other commonly used classification techniques when confidence intervals are considered.

Classifier		Accuracy	AUROC
N-UFA	UFA-based	77.5% (75.1, 79.9)	0.819 (0.797, 0.841)
RF-UFA		78.1**%** (75.8, 80.3)	0.800 (0.779, 0.821)
Logistic Regression	Other	68.7% (65.7, 71.6)	0.698 (0.642, 0.753)
Support Vector Machine		79.4% (76.2, 82.6)	0.555 (0.331, 0.780)
Decision Tree		68.8% (66.0, 71.7)	0.626 (0.575, 0.677)
Random Forest		79.0% (76.9, 81.1)	0.823 (0.796, 0.851)

To further test UFA’s ability to make predictions in the presence of missing and noisy data, we artificially introduced additional missing values and random deviances into the MIMIC II data.

Table 6 compares the performance of N-UFA, random forest, and logistic regression for the original MIMIC II data and a version of the MIMIC II data where 50% of observations were replaced randomly with missing values. While N-UFA and random forest performed similarly on the original MIMIC II dataset (Table 5), we see that N-UFA has more consistent performance as the amount of missing data increases. Table 6 shows that the difference in accuracy between no additional missing data and 50% additional missing data for the N-UFA approach is only 1.3 percentage points, compared to 7.1 percentage points for random forest. Similarly, AUROC decreases by 0.029 as opposed to 0.052.

**Table 6:** Comparison of different classifiers with varying amounts of missing data. This table compares the performance of different classifiers for the original MIMIC II data and a version of the MIMIC II data where 50% of observations were replaced randomly with missing values. We see that N-UFA is robust to missing data, with accuracy decreasing just 1.3% as the amount of missing data increases to 50%. An expanded version of Table 6 including confidence intervals and results for 5–25% missing data is available in the S1 Table.

Classifier		Accuracy			AUROC		
		0%	50%	Δ	0%	50%	Δ
N-UFA	UFA-Based	77.5%	76.2%	**1.3%**	0.819	0.790	**0.029**
Random Forest	Other	79.0%	71.9%	**7.1%**	0.823	0.771	**0.052**
Logistic Regression		68.7%	58.3%	**10.4%**	0.698	0.598	**0.100**

Table 7 provides similar results for data accuracy. It presents accuracy and AUROC for N-UFA, random forest, and logistic regression when 50% of the MIMIC II data is randomly perturbed by a value *ϵ*, distributed normally with mean zero and the empirical variance of the variable in question. The results show that all three methods hold up well to the introduction of random noise.

**Table 7:** Comparison of different classifiers with varying amounts of imprecise data. This table compares the performance of different classifiers for the original MIMIC II data and a version of the MIMIC II data where 50% of observations were randomly perturbed by a value *ϵ*, distributed normally with mean zero and the empirical variance of the variable in question. We see that N-UFA is robust to imprecise data, with accuracy decreasing 1.7% as the amount of imprecise data increases to 50%. An expanded version of Table 7 including confidence intervals and results for 5–25% imprecise data is available in the S2 Table.

Classifier		Accuracy			AUROC		
		0%	50%	Δ	0%	50%	Δ
N-UFA	UFA-Based	77.5%	75.8%	**1.7%**	0.819	0.796	**0.023**
Random Forest	Other	79.0%	76.3%	**2.7%**	0.823	0.802	**0.021**
Logistic Regression		68.7%	68.8%	**-0.1%**	0.698	0.681	**0.017**

### Rare events (landslide data)

In our last application, we use UFA to predict the rare event of landslides. This dataset contains all reported landslides for Seattle, WA from 1965 to 1999 [28]. Each observation represents one day, and contains information on precipitation, temperature, and wind, along with whether a landslide was reported. Of the nearly 13,000 days in the dataset, only 2.3% had one or more landslide. Given the relative infrequency of the target, we focused our analysis on identifying variables or groups of variables that were predictive of an increased risk.

**Fig 3:**
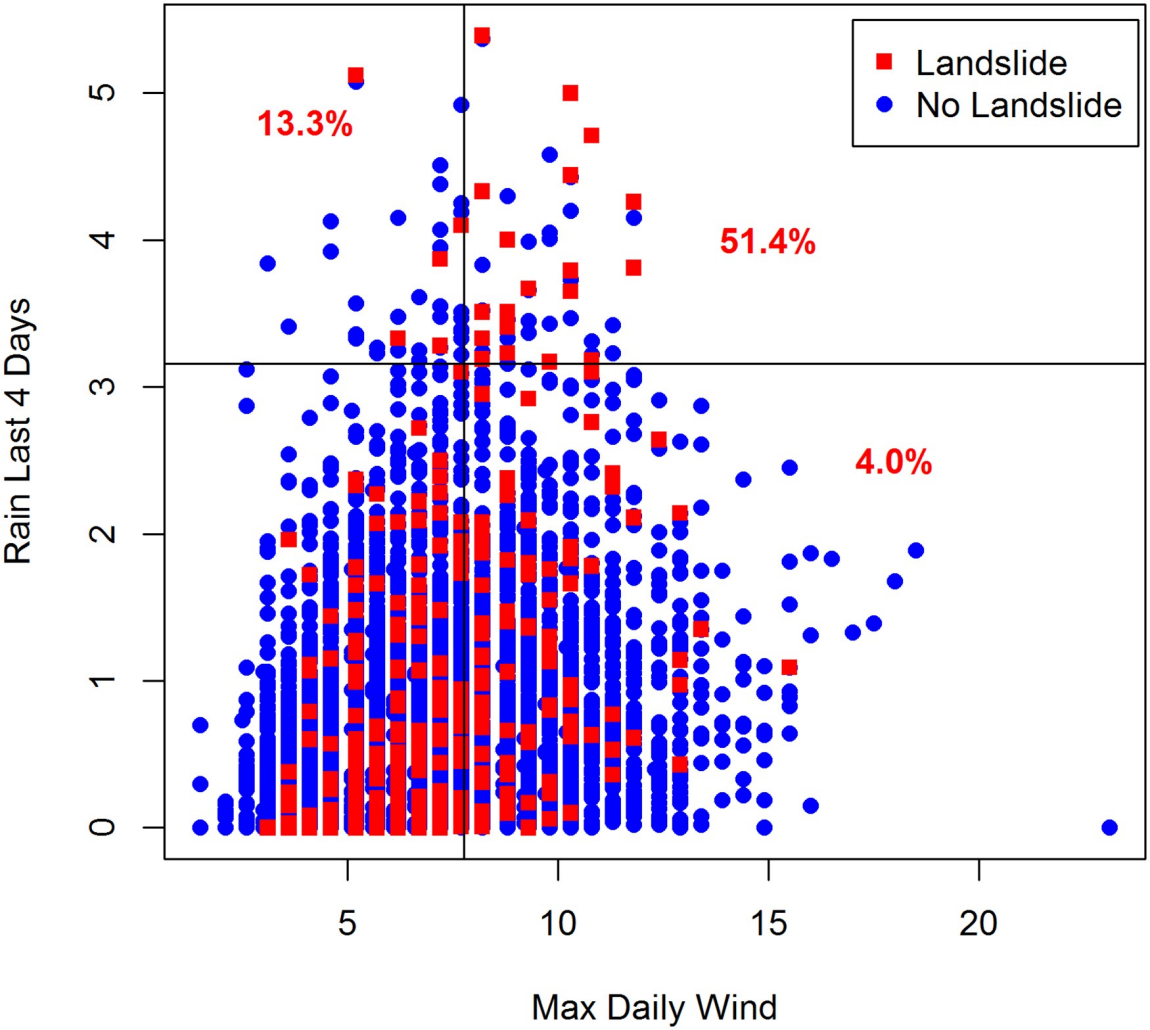
Landslide days stratified by precipitation and wind. The percentage of days with a landslide in each quadrant is displayed in red. Thresholds can be combined to find conditions under which the relative risk of a landslide is significantly elevated.

UFA identified 32 significant thresholds associated with an increased likelihood of landslide. For example, when precipitation for the last four days exceeds 3.2 inches, the percentage of days with a landslide is 34.3%, nearly 15x the rate for a typical day. Moreover, as seen in Fig 3, thresholds can be combined to find conditions under which the relative risk of a landslide is even higher. If one combines the rain threshold with a maximum daily wind of more than 7.8, the percentage of days with a landslide jumps to 51.4%, more than 22x the rate for a typical day.

In general, it is difficult to train classifiers when the incidence of the target is very low [29]. This is because the classifier can achieve very high accuracy by always predicting the more likely outcome; in this case, a model that always predicts no landslide would have an accuracy of 97.7%. One solution is to balance the training dataset, so that it has an equal number of days with and without a landslide. However, this may be undesirable for a variety of reasons. In particular, for very rare events, balancing the dataset through under sampling may exclude a large number of potentially useful majority-class examples, while balancing the dataset through oversampling can lead to overfitting [30].

We find that one advantage of N-UFA is that it can identify days that are at high risk of landslide, even with unbalanced training data. Using 80% of our landslide dataset for training, we find that days with 12 or more flags are 14.8x more likely to have a landslide. If we consider this the definition of a “high risk” day, and apply the same criteria to the remaining 20% of the data, we see that this definition generalizes well. The rate of landslides on high risk days in the test set is 18.2%, almost 8x the typical rate.

## Discussion

Donoho and Jin [1] used a set of standard medical datasets proposed by Dettling [2] for evaluation of machine learning algorithms to demonstrate that their very simple ‘clipping rule’ algorithm performed as well as most commonly used algorithms, including Regression, Decision Tree, Random Forest, SVM, and KNN. Our analysis suggests that further analysis of univariate approaches is warranted, and, if accurate enough, these approaches have a number of advantages when compared to other methods.

UFA automatically detects target-defined separation thresholds in data. In the results section, we showed that the thresholds that it detects for variables for which such boundaries are known align with published results and scientifically established boundaries and can be used to classify previously unseen data with performance equal to or better than other standard classifiers. We also showed that the UFA system works well under certain challenging conditions that often arise with big data.

Also, since UFA runs on each variable individually, it can easily be applied to datasets with a very large number of features, including cases when the number of features is much larger than the number of observations. Thresholds for each variable can be identified in parallel, allowing for efficient computing. Further, though UFA is univariate, the ability to quickly and automatically consider a large number of features means that researchers can easily introduce new variables that are interactions of existing features and evaluate whether they are important for the application. For example, in the case of the Golub gene expression data, UFA was able to identify approximately 1,200 meaningful thresholds/variables from a set of 7,129 without any preprocessing which is in line with Golub’s original findings [20].

UFA extracts a clear decision rule for each variable that is identified as significant. These rules take the simple form of:

For [*variable*], a value [*above/below*] [*threshold*] is associated with a significantly [*higher/lower*] incidence of outcome [*Y*]

Thresholds of this type are easy to understand, interpret, and verify against pre-existing domain knowledge, giving UFA a high level of interpretability. Classification is straightforward as well. As introduced in this paper, the N-UFA classifier distills information from potentially thousands of independent variables to two dimensions, making results easy to visualize. One can simply create a plot with high risk flags on one axis and low risk flags on the other, along with the relevant decision boundary (example in Fig 2). New cases can then be added to the same plot, and the user can easily see where the instance falls both in terms of its classification, as well as its distance from the decision boundary.

Furthermore, N-UFA maintains this high level of interpretability and simplicity without compromising predictive performance. For benchmark, MIMIC II, and landslide data, N-UFA performed comparably to the state-of-the-art methods. However, in most cases it is easier to interpret simple univariate rules than understand more complex relations, e.g rules coming from Random Forest. Random Forest classifier averages over a large number of trees, which may utilize different rules. In addition, while there are standard visualizations for Random Forest, such as variable importance plots and proximity plots, it has been observed that proximity plots tend to look similar across different datasets, leading some to question their utility [5]. Therefore, N-UFA may be a preferable approach for certain applications where visualization of results is required.

The UFA system does not require complete data, which makes it easy to implement on data with a large number of missing values. If an observation is missing data for a particular variable, it can simply be excluded from the calculation of that variable’s threshold, but remain included in calculations for which data are present. This gives UFA an advantage over classifiers that require complete data for prediction, as those classifiers must impute data or ignore incomplete observations, significantly decreasing the amount of data available for analysis.

Formal specification of conditions under which the UFA system breaks down was outside the scope of this paper. However, our empirical results show that N-UFA has performance equal to or better than other standard classifiers on the MIMIC II data, a real-world dataset where nearly 65% of patients have incomplete data. Furthermore, the results show that N-UFA is comparable to or better than other classifiers as the amount of missing data is increased (Tables 3 and 6).

Another challenge often observed in large, real-world datasets is a low target/non-target ratio. However, there is often a high level of interest in predicting atypical events, such as rare disease, or an unusual disease outbreak. As demonstrated using the Seattle landslide data, the UFA system can easily be used to focus on variables that increase the relative risk of a rare event. In fact, we found that N-UFA could successfully identify days with a high likelihood of landslide even when the training data were unbalanced.

One of the limitations of the UFA system is that it conducts *nk* statistical tests in the training phase in order to identify the optimal thresholds, where *n* is the number of variables and *k* is the number of potential thresholds. As is well documented, multiple hypothesis testing can inflate the type I error rate and lead to significant results, even when none exist [8, 5]. This drawback is also present in related methods, such as the minimum p-value approach to finding optimal cut points, and a variety of solutions have been suggested. In this paper, we address the issue through validating the thresholds on previously unseen data. Another possibility is to adjust the p-values in the training phase for multiple testing, using an approach such as the well-known Bonferroni method [5] or the false discovery rate [31], though these approaches were not explored here.

## Conclusion

This paper presents a simple algorithm for identifying univariate thresholds in data. Following the approach proposed by Donoho and Jin [1] and Leskovec, et al. [32] we demonstrate that the univariate approach to the analysis of clinical data achieves similar predictive accuracy compared to more sophisticated machine learning algorithms, while providing easily interpretable results and stability against missing data without the need for data imputation, a highly undesirable operation in clinical data analysis. UFA builds on previous work in this area by only considering a subset of the input space, while simultaneously being fully automated.

The thresholds generated by UFA can easily be combined to predict outcomes for previously unseen cases. Though a variety of methods for combining the thresholds exist, in this paper, we introduce N-UFA which classifies observations based on their number of high risk and number of low risk flags. N-UFA greatly reduces the dimensionality of the problem and has similar or better performance than many other commonly used classification techniques, including random forest when confidence intervals are considered. In addition to strong predictive performance, this paper highlights several other key advantages of the UFA:

1. Fully automated; can be used with little a priori knowledge of the data
2. Scales to a large number of variables, even if the number of variables exceeds the number of observations
3. Provides the user with simple rules characterizing relationship between individual variables and the outcome
4. Stable against noise and missing data
5. Useful when the incidence of the target is low
6. Displays results in two dimensions making it easy to interpret and visualize

Future work should focus on better capturing the uncertainty inherent to the thresholds, potentially through methods such as bootstrapping. Future work is also needed to formalize the conditions in which the UFA system performs well, which are explored empirically in this initial paper.

## Supporting information

S1 Table Comparison of different classifiers with varying amounts of missing data with confidence intervals. S1 Table compares the performance of the number of flags classifier to random forest, and logistic regression. For each row of the table an increasing percentage of each variable in the MIMIC II dataset was randomly replaced with missing values.
(PDF)

S2 Table Comparison of different classifiers with varying amounts of noisy data with confidence intervals. S2 Table compares the same three classifiers for varying amounts of imprecise data. For each row, an increasing percentage of each variable in MIMIC II is randomly perturbed by a value ϵ, distributed normally with mean zero and the empirical variance of the variable in question.
(PDF)
