# Supplementary material for: The Univariate Flagging Algorithm (UFA): An interpretable approach for predictive modeling
Source: PLoS One. 2019 Oct 11;14(10):e0223161. doi: 10.1371/journal.pone.0223161 (PMC6788700; doi:10.1371/journal.pone.0223161)
Supplement: S1 Table — compares the performance of the number of flags classifier to random forest, and logistic regression. For each row of the table an increasing percentage of each variable in the MIMIC II dataset was randomly replaced with missing values. (PDF) [file pone.0223161.s001.pdf]

% Missing	Number of Flags		Random Forest		Logistic Regression	
	UFA-based		Original data		Original data	
	Accuracy	AUC	Accuracy	AUC	Accuracy	AUC
0%	77.5% (75.1, 79.9)	0.819 (0.797, 0.841)	79.0% (76.9, 81.1)	0.823 (0.796, 0.851)	69.7% (65.7, 71.6)	0.698 (0.642, 0.753)
5%	77.5% (74.9, 80.1)	0.820 (0.793, 0.847)	78.3% (76.7, 79.8)	0.812 (0.783, 0.841)	68.5% (67.2, 69.8)	0.659 (0.644, 0.673)
10%	78.1% (75.3, 80.8)	0.817 (0.793, 0.842)	77.1% (73.6, 80.7)	0.812 (0.785, 0.840)	66.0% (63.1, 68.8)	0.636 (0.600, 0.672)
25%	77.9% (76.0, 79.7)	0.816 (0.792, 0.839)	76.9% (74.7, 79.1)	0.819 (0.791, 0.847)	67.5% (64.1, 70.9)	0.631 (0.576, 0.686)
50%	76.2% (73.9, 78.4)	0.790 (0.764, 0.815)	71.9% (69.3, 74.6)	0.771 (0.744, 0.799)	58.3% (53.3, 63.2)	0.598 (0.566, 0.629)
