# Supplementary material for: The Univariate Flagging Algorithm (UFA): An interpretable approach for predictive modeling
Source: PLoS One. 2019 Oct 11;14(10):e0223161. doi: 10.1371/journal.pone.0223161 (PMC6788700; doi:10.1371/journal.pone.0223161)
Supplement: S2 Table — compares the same three classifiers for varying amounts of imprecise data. For each row, an increasing percentage of each variable in MIMIC II is randomly perturbed by a value ϵ, distributed normally with mean zero and the empirical variance of the variable in question. (PDF) [file pone.0223161.s002.pdf]

% Varied	Number of Flags		Random Forest		Logistic Regression	
	UFA-based		Original data		Original data	
	Accuracy	AUC	Accuracy	AUC	Accuracy	AUC
0%	77.5% (75.1, 79.9)	0.819 (0.797, 0.841)	79.0% (76.9, 81.1)	0.823 (0.796, 0.851)	68.7% (65.7, 71.6)	0.698 (0.642, 0.753)
5%	77.3% (74.5, 80.2)	0.808 (0.785, 0.830)	76.2% (74.0, 78.3)	0.805 (0.777, 0.833)	65.4% (61.7, 69.1)	0.638 (0.602, 0.674)
10%	76.5% (73.7, 79.3)	0.811 (0.788, 0.834)	77.5% (74.2, 80.8)	0.816 (0.782, 0.851)	70.0% (68.1, 71.9)	0.694 (0.671, 0.717)
25%	77.9% (75.1, 80.6)	0.811 (0.786, 0.836)	77.1% (74.9, 79.3)	0.795 (0.768, 0.822)	63.1% (59.1, 67.1)	0.611 (0.563, 0.658)
50%	75.8% (73.0, 78.5)	0.796 (0.766, 0.825)	76.3% (73.7, 79.0)	0.802 (0.775, 0.829)	68.8% (64.6, 73.1)	0.681 (0.635, 0.727)
